# Transglutaminase 2 regulates endothelial cell calcification via IL-6-mediated autophagy

**DOI:** 10.3389/fphar.2024.1393534

**Published:** 2024-11-25

**Authors:** Bo Liu, Zhiyuan Cai, Yan Wang, Xinye Liu, Bin Zhang, Qian Zheng, Jingye Li, Cien Li, Yuanbo Cui, Pengju Lv, Dongwei Yang

**Affiliations:** ^1^ Department of Cardiology, Zhengzhou Central Hospital, Zhengzhou University, Zhengzhou, Henan, China; ^2^ The First Department of Ocular Fundus Diseases, Zhengzhou Second Hospital, Zhengzhou, Henan, China; ^3^ Translational Medical Center, Zhengzhou Central Hospital, Zhengzhou University, Zhengzhou, Henan, China; ^4^ Department of clinical laboratory, Zhengzhou Central Hospital, Zhengzhou University, Zhengzhou, Henan, China

**Keywords:** transglutaminase 2, calcification, autophagy, interleukin-6, autocrine, endothelial cells

## Abstract

**Introduction:**

Endothelial cell (EC) calcification is an important marker of atherosclerotic calcification. ECs play a critical role not only in atherogenesis but also in intimal calcification, as they have been postulated to serve as a source of osteoprogenitor cells that initiate this process. While the role of transglutaminase 2 (TG2) in cellular differentiation, survival, apoptosis, autophagy, and cell adhesion is well established, the mechanism underlying the TG2-mediated regulation of EC calcification is yet to be fully elucidated.

**Methods:**

The TG2 gene was overexpressed or silenced by using siRNA and recombinant adenovirus. RT-PCR and WB were used to analyze the relative expression of target genes and proteins. 5-BP method analyzed TG2 activity. mCherry-eGFP-LC3 adenovirus and transmission electron microscopy analyzed EC autophagy level. Calcium concentrations were measured by using a calcium colorimetric assay kit. Alizarin red S staining assay analyzed EC calcification level. Elisa analyzed IL-6 level. Establishing EC calcification model by using a calcification medium (CM).

**Results:**

Our findings demonstrated that CM increased TG2 activity and expression, which activated the NF-κB signaling pathway, and induced IL-6 autocrine signaling in ECs. Furthermore, IL-6 activated the JAK2/STAT3 signaling pathway to suppress cell autophagy and promoted ECs calcification.

**Discussion:**

ECs are not only critical for atherogenesis but also believed to be a source of osteoprogenitor cells that initiate intimal calcification. Previous research has shown that TG2 plays an important role in the development of VC, but the mechanism by which it exerts this effect is not yet fully understood. Our results demonstrated that TG2 forms complexes with NF-κB components inhibition of autophagy promoted endothelial cell calcification through EndMT. Therefore, our research investigated the molecular mechanism of EC calcification, which can provide new insights into the pathogenesis of atherosclerosis.

## 1 Introduction

Vascular calcification (VC) commonly occurs in aging, atherosclerosis, hypertension, diabetes, dyslipidemia, cardiac valve disease, and chronic kidney disease, which is caused by the ectopic deposition of hydroxyapatite (basic calcium phosphate) on the vascular wall (Madhavan et al., 2014). Growing evidence suggests that VC is closely related to the metabolism of bone development and cartilage formation, and is an active, multifactorial, and biologically regulated physiological process (Bostrm, 2016). Intimal calcification, also known as atherosclerotic calcification, is more commonly observed in certain large arteries, such as the aorta and carotid arteries, and it affects the arterial lumen and smoothness of blood flow (Ryu et al., 2021). Endothelial cells (ECs) can undergo a transdifferentiation process named endothelial-to-mesenchymal transition (EndMT) to become pluripotent. They are committed to osteogenic differentiation guided by various stimuli, including bone morphogenetic proteins (BMPs) (Sánchez-Duffhues et al., 2019). For example, oxidized low-density lipoprotein induced oxidative stress in ECs in a BMP6-dependent manner, consistent with prior observations that BMP2 induced calcification in cultured ECs in an oxidative stress-dependent manner (Yung et al., 2015). Autophagy is an evolutionarily conserved process that sequesters nonessential intracellular components for lysosomal degradation in response to various stress stimuli, including nutrient or growth factor deprivation, hypoxia, reactive oxygen species, DNA damage, protein aggregates, damaged organelles, and intracellular microorganisms (Levine and Kroemer, 2008). Autophagy plays a significant role in VC, as it can reduce electrolyte imbalance while regulating stromal vesicle release and promoting or inhibiting VC, depending on the pathophysiological context (Zhou et al., 2021). Additionally, autophagy can inhibit or induce vascular endothelial inflammation, which is a key factor in the development of VC (Wang et al., 2019; Vion et al., 2017; Wu et al., 2019). Transglutaminase type 2 (TG2) is a multifunctional, ubiquitously expressed member of the TG family that catalyzes the post-translational modifications of proteins through Ca^2+^-dependent and Ca^2+^-independent reactions (Rossin et al., 2018). TG2 is distinguished from other transglutaminases by its ubiquitous expression, widespread localization, and ability to bind to and hydrolyze guanine nucleotides (Rossin et al., 2018). Owing to its multi-functionality, TG2 has been reported to have a complex biology that plays a role in a variety of cellular processes, such as differentiation, survival, apoptosis, autophagy, and cell adhesion (D'Eletto et al., 2018). Previous research has shown that TG2 plays an important role in the development of VC, but the mechanism by which it exerts this effect is not yet fully understood (Johnson et al., 2008). IL-6 is a multifunctional cytokine secreted by T cells, macrophages, ECs, and other cells, thereby playing a key role in regulating the immune response and promoting inflammation. A study on cultured human valve interstitial cells (VICs) found that exposure to high Pi-activated NF-kB levels leads to increased IL-6 secretion. Specifically, Pi-induced mineralization was largely dependent on IL-6 expression because blocking IL-6 expression using small interfering RNA (siRNA) decreased calcification in VICs (Henaut et al., 2016). This suggests that IL-6 plays a crucial role in the calcification process in VICs and is a potential target for preventing or treating VIC calcification. The JAK/STAT molecular pathway is activated by a broad range of profibrotic and pro-inflammatory cytokines, including IL-6, IL-11, and IL-13 (Montero et al., 2021). The signal transducer and activator of transcription (STAT3) is a key protein in the JAK/STAT signaling pathway, which was initially identified as a transcriptional enhancer of acute phase genes that are activated by IL-6. Recent studies have also implicated STAT3 in various aspects of the autophagic process (Montero et al., 2021; You et al., 2015). Current research on the pathogenesis of VC has primarily focused on vascular smooth muscle cells (VSMCs); however, EC calcification is closely related to atherosclerotic calcification. To better understand the underlying mechanisms of atherosclerotic calcification, our study used human umbilical vein ECs (HUVECs) to establish an EC calcification model and investigate the role of TG2 in regulating EC calcification, thereby shedding light on new therapeutic targets for preventing or treating atherosclerotic calcification.

## 2 Materials and methods

### 2.1 Reagents

For cell culture, Dulbecco’s modified Eagle’s medium, penicillin, and streptomycin were purchased from Pricella, Procell Life Science & Technology, China; fetal bovine serum was bought from Lonsa Science, Uruguay; and trypsin-EDTA (0.25%) phenol red was obtained from Gibco, Thermo Fisher Scientific, United States. For calcification induction, BGP, dexamethasone, ascorbic acid, GK921, and 5-biotinamidopentylamine (5-BP) were obtained from Merck KGaA, Germany, whereas BMP-2 protein was purchased from MedChemExpress, United States. Meanwhile, for autophagy inhibition and induction, Chloroquine phosphate(CQ), AZD-1480 and IL-6 protein were acquired from MedChemExpress, United States, mCherry-eGFP-LC3 was purchased from Hanbio Tech China, and siRNA transfection reagent was obtained from Polyplus technology, French. The following fixation and staining reagents were used in this study: paraformaldehyde (4%), glutaraldehyde (2.5%, EM Grade), and Triton-X100 (Solarbio LIFE SCIENCES, China); alizarin red solution (OriCell, China); and 4′,6-diamidino-2-phenylindole (DAPI) and streptavidin/AF555 (Bioss antibodies, China). In addition, for protein analysis, the following reagents and antibodies were used: RIPA lysis solution (Epizyme Biotech, China), phosphatase inhibitor cocktail (Epizyme Biotech, China), multicolor prestained protein ladder (Epizyme Biotech, China), protein sample loading buffer (Epizyme Biotech, China), anti-LC3B antibody (CST, United States, diluted 1:1,000), anti-TG2 antibody (Abcam, United States, diluted 1:1,000), anti-RunX2 (Abcam, United States, diluted 1:1,000), anti-BMP2 (Abcam, United States, diluted 1:1,000), anti-GAPDH (Abcam, United States, diluted 1:1,000), NF-ĸB1 (Abcam, United States, diluted 1:1,000), P65 (Abcam, United States, diluted 1:1,000), STAT3 (Protein Tech, China, diluted 1:1,000), anti-p-P65 (phospho-S536, Abcam, United States, diluted 1:1,000), anti-p-JAK1 (phospho-Y1022, Bioworld Technology, China, diluted 1:500), anti-p-JAK2 (Phospho-Y1007/1,008, Bioworld Technology, China, diluted 1:500), anti-p-STAT3 (phospho-S727, Bioworld Technology, China, diluted 1:500), anti-JAK1/JAK2 (Bioworld Technology, China, 1:500), STAT3(Protein Tech, China,1:500), CD44(Bioworld Technology, China,1:500). Finally, for molecular analysis, the following reagents were used: RNA easy fast tissue/cell kit, fast king kit (with gDNase), and talent qPCR premix (SYBR Green; TIANGEN Biotech, China).

### 2.2 Cells and treatments

A normal HUVEC line was purchased from Procell Life Science and Technology Co., Ltd. (Wuhan, China). To establish the EC calcification model, HUVECs were treated with a calcification medium (CM) consisting of 10% FBS, 100 nM dexamethasone, 0.2 mM ascorbic acid, 20 mM β-glycerolphosphate, and 200 ng/mL BMP-2 for 14 days (Yung et al., 2015; Kim et al., 2012; Kim et al., 2021). The CM was changed every 3 days.

### 2.3 siRNA and recombinant adenovirus

To perform siRNA and recombinant adenovirus transfections in HUVECs, cells were used at 70%–80% confluency in six-well plates. siRNA transfection was performed by mixing 2 μL INTERFERin^®^ siRNA transfection reagent (Polyplus technology, French) with 4 μL siRNA in 400 μL serum-free and antibiotic-free DEMEM/F-12 medium. siRNA and INTERFERin^®^ siRNA transfection reagent were mixed thoroughly using lab-dancer and incubated for 10 min at room temperature. The resulting siRNA–lipid complex was added to HUVECs with 1,600 µL complete medium, which was replaced after 12 h. After transfection for 48 h, cells were collected and the efficiency of gene silencing was determined using Western blot. Recombinant adenovirus (1 × 10^10^ TU/mL) was diluted 1,000 times in complete medium and added to HUVECs in a six-well plate (1 mL/well). The medium was replaced with fresh complete medium after 12 h. HUVECs were cultured for 72 h, and the efficiency of gene overexpression was determined using Western blot. Subsequently, following stable transfection, HUVECs were treated with CM. siRNA targeting human TG2 mRNAs were purchased from GenePharma Biotech Company, China, with the following sequences: TG2 siRNA: sense strand: GCC​UGA​UCC​UUC​UAG​AUG​UTT; antisense strand: ACA​UCU​AGA​AGG​AUC​AGG​CTT, NC-siRNA: sense strand: UUCUC CGAACGUGUCUTT; anti sense strand: ACGUGACACGUU CGGAGA ATT. For TG2 overexpression, human recombinant adenoviral vectors were purchased from GenePharma Biotech Company, China, and the negative control was the empty virus.

### 2.4 TG2 activity assay

To measure intracellular TG2 activity, 5-BP incorporation was visualized using fluorochrome-conjugated streptavidin HRP (Penumatsa et al., 2014). In brief, after culture in 24-well plates, HUVECs were serum-starved overnight, pretreated with 5-BP (400 μM) for 4 h, and then treated with either GK921 (250 nM) or vehicle control for 24 h. For negative control, 5-BP incubation was omitted. After a brief wash with PBS, cells were fixed with 4% formaldehyde and permeabilized with 0.2% Triton X-100 in PBS. Permeabilized cells were then blocked with 5% BSA in PBS and incubated with Streptavidin AlexaFluor/AF 555 HRP conjugate for 4 h in a blocking buffer. The nuclei were stained with DAPI for 5 min and then washed with PBS three times. The DAPI-stained cells were imaged using Olympus light microscope (BX43F) and LAS V4 software. TG2 activity was quantified by measuring the intensity of 5-BP staining per cell.

### 2.5 RT-PCR

The RNAprep Pure Cell Kit (TianGen BioTech, China) was used to isolate total RNA from HUVECs with various treatments according to the manufacturer’s protocol. The RNA concentration and purity were determined using NanoDrop 2000 spectrophotometer (Thermo Scientific). Equal amounts of total RNA were reverse-transcribed using the FastQuant RT Kit (with gDNase) (TianGen BioTech, China), and RT-PCR was performed with the SuperReal PreMix Plus (SYBR Green) (TianGen BioTech, China) and 7,500 Fast Dx Real-time PCR Instrument (Applied Biosystems; Thermo Fisher Scientific, United States). The primers used were as follows (Hu et al., 2020; Ingwersen et al., 2022):

hTG2-s TAT​GGC​CAG​TGC​TGG​GTC​TTC​GCC and h-TG2-a GGCTCC AGG​GTT​AGG​TTG​AGC​AGG;

h-BMP-2-s GCG​TGA​AAA​GAG​AGA​CTG​CG and h-BMP-2-a ACCAT GGTCGACCTTTAGGAG;

h-RunX2-s CGC​CTC​ACA​AAC​AAC​CAC​AG and h-RunX2-a ACTGC TTGCAGCCTTAAATGAC;

h-GAPDH-s CAG​GGC​TGC​TTT​TAA​CTC​TGG​T and h-GAPDH-a GATT TTGGAGGGAT CTGGCT.

The relative gene expression values were calculated using the ΔΔCt method (Ct = ΔΔCt treated − ΔΔCt untreated control), with the housekeeping gene GAPDH as an internal control. The fold change in the mRNA level of each gene was calculated using the 2^−ΔΔCT^ method.

### 2.6 Western blot analysis

Cells were collected from a 6-well plate by adding 200 μL RIPA lysate to each well. In brief, protein samples were loaded at a concentration of 30 μg per lane and separated using 10% or 12.5% SDS-PAGE gels. The separated proteins were then transferred onto polyvinylidene fluoride or polyvinylidene difluoride (PVDF) membranes. Following the transfer, the membranes were cut horizontally into small segments according to a multicolor prestained protein ladder (Epizyme Biomedical Technology, China) and protein molecular weight. Proteins with similar molecular weight were transferred separately. The cut membranes were incubated overnight at 4°C with the anti-TG2 monoclonal antibody, anti-RunX2 polyclonal antibody, anti-BMP2 polyclonal antibody, anti-LC3B polyclonal antibody, anti-NF-κB1 polyclonal antibody, anti-P65/p-P65 polyclonal antibody, anti-JAK1/p-JKA1 polyclonal antibody, anti-JAK2/p-JKA2 polyclonal antibody, anti-STAT3/p-SAT3 polyclonal antibody, and anti-GAPDH monoclonal antibody. Membranes were subsequently washed and incubated with an HRP-conjugated secondary antibody, followed by the detection of bands using the Omni-ECL™ Femto Light Chemiluminescence Kit (Epizyme Biomedical Technology, China). The chemiluminescent image was captured using the BIO-RAD ChemiDoc™ XRS + Chemiluminescent Imaging System with image Lab™ software for 1–3 min. The bands of interest were identified based on their apparent molecular sizes. The target protein levels were normalized relative to GAPDH levels and expressed as a fold change relative to the original group. Image J-Pro Plus 6 software was used to quantify the intensity of the immunoreactive bands of interest on autoradiograms.

### 2.7 mCherry-eGFP-LC3 adenovirus transfection

HUVECs at 50% confluence in a 24-well plate were transfected with the mCherry-eGFP-LC3 recombinant adenovirus (mCherry-eGFP-LC3). mCherry-eGFP-LC3 was diluted in a complete medium to a concentration of 10^6^ pfu/mL and added to the cells according to the instructions. Three replicates were established for each group, and medium was replaced after 24 h. Cells were then cultured at 37°C with 95% air and 5% CO_2_ for another 24 h and fixed with 4% paraformaldehyde for 30 min at room temperature. The nuclei were stained with DAPI for 5 min and then washed with PBS three times. Next, the cells were protected from light, and images were captured using a fluorescence microscope (BX43F, Olympus, Japan).

### 2.8 Transmission electron microscopy

HUVECs were inoculated in 6-well plates at a density of 8 × 10^4^ cells/mL per well. Three replicates were used for each group with different reagents and/or chloroquine phosphate, IL-6 and AZD-1480 to culture cells for 24 h. After fixation with glutaraldehyde, cells were stored at 4°C until further use. HUVECs were observed using transmission electron microscopy after fixation. After re-fixation with osmium tetroxide, cells were subjected to gradient dehydration, followed by drying in a supercritical extraction apparatus. The dried cells were then sprayed with gold, attached to a sample stage, and observed using a transmission electron microscope (Leica, HT7800/HT7700, Germany). The number of autophagosomes per cytoplasmic area was quantified by counting 3–5 cells per sample according to the reference. (Cai et al., 2020; Hu et al., 2021).

### 2.9 Alizarin red S staining assay

HUVECs were inoculated in 24-well plates at a cell density of 1 × 10^6^ cells/mL. Fixation was performed in 4% paraformaldehyde for 15 min. The fixative was discarded, and cells were washed with PBS three times. Then, alizarin red S solution (2%) was added gradually and incubated for 30 min at room temperature. The dye was removed, and cells were washed five times with PBS. To prevent drying, 500 µL PBS was added to each well. HUVECs were then visualized under a microscope (BX43F, Olympus, Japan). The calcification sites were orange–red in color.

### 2.10 Quantification of calcium deposition

HUVECs were inoculated in 6-well plates at a density of 8 × 10^4^ cells/mL per well, and calcium concentrations were measured using a calcium colorimetric assay kit (Beyotime Biotechnology, China) (Zhao et al., 2021; Cheng et al., 2020). In brief, cells were washed twice with PBS and then incubated with 0.6 M HCL for 24 h. Working solution (200 µL) was added to each well, and the cells were incubated at 37°C for 5 min. After extensive lysis, the cells were centrifuged at 14,000 × g for 5 min at 4°C and the supernatant was collected and placed on ice. The BCA assay (Beyotime Biotechnology, China) was used to measure the protein content, and the concentration of each sample was adjusted for consistency. Meanwhile, 0, 0.1, 0.2, 0.4, 0.6, 0.8- and 1.0-mM calcium standards were prepared according to manufacturer’s instructions. Approximately 50 µL standard or sample was added to each well of a 96-well plate, and each concentration standard and sample was detected in two duplicate wells. Finally, absorbance at 575 nm was measured using the SpectraMax M2 spectrometer (Molecular Devices, Sunnyvale, CA, United States).

### 2.11 Enzyme linked immunosorbent assay (ELISA)

HUVECs were seeded in 96-well plates (5 × 10^3^ cells/well, 200 μL). siRNA and adenovirus preparation followed the same procedure as that described previously. After incubation, 50 μL of cell supernatant was collected from each well, and the samples were centrifuged for 20 min at 1,000 × g. The supernatant was collected and either immediately analyzed or stored in an aliquot at −80°C for later use. Total IL-6 in the supernatant with different treatments was quantified using the High Sensitive ELISA Kit for IL-6 (Cloud-Clone Corp., Wuhan, China) according to the manufacturer’s instructions. A microplate reader (SpectraMax®M2e, Molecular DEVICES, United States) was used to measure absorbance at 450 nm.

### 2.12 Statistical analysis

All experiments were performed in triplicate, and data are expressed as the mean ± standard deviation. Statistical analysis was performed using the GraphPad Prism 6.01 and SPSS 22.0 software. Student’s t-test and one-way analysis of variance were used to analyze the differences between means. A *p*-value of <0.05 was considered statistically significant.

## 3 Results

### 3.1 Establishment of EC calcification model

**FIGURE 1:**
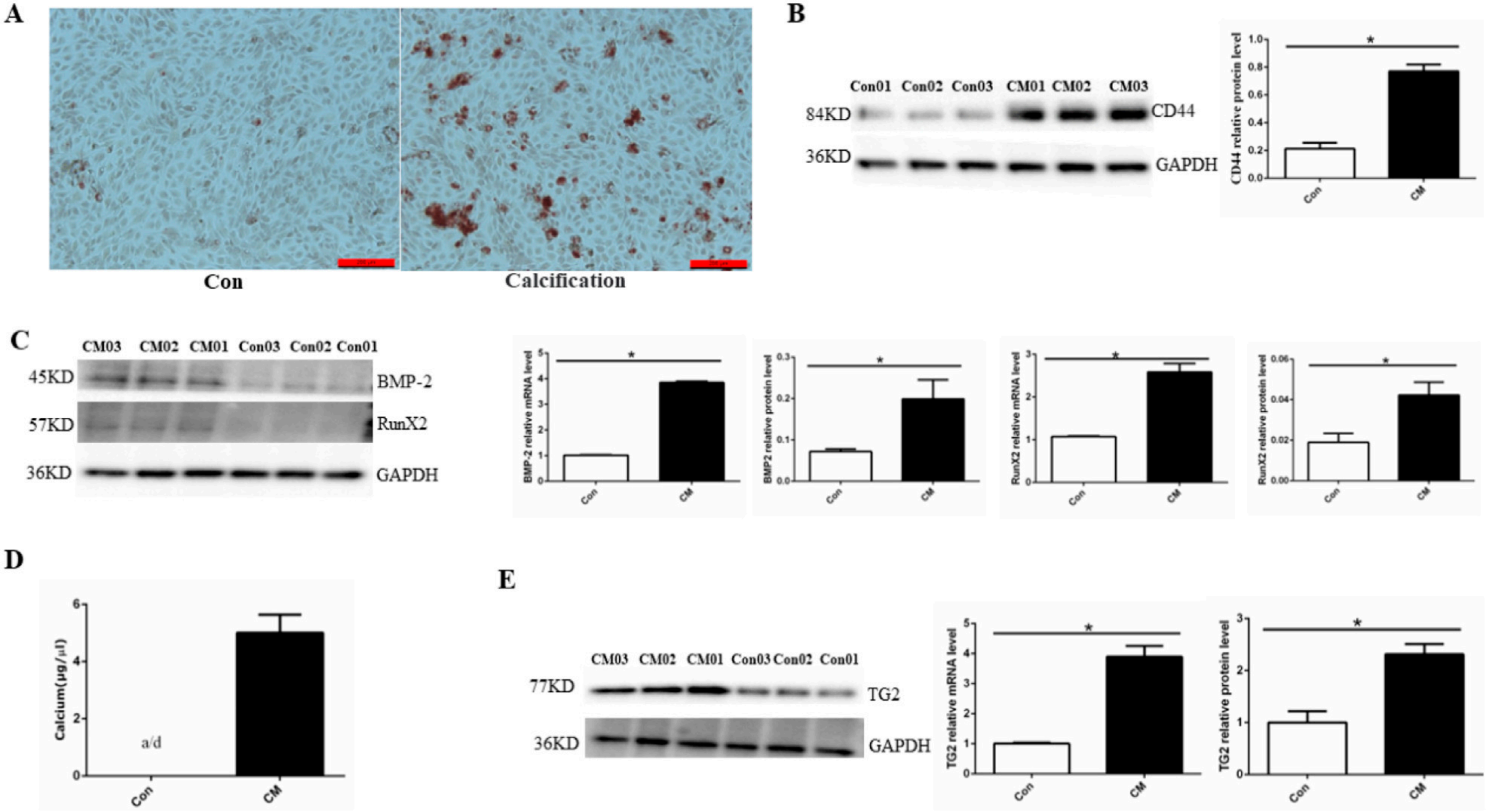
Establishing a HUVEC calcification model. **(A)** Alizarin red S staining detects calcium salt deposits, with an orange–red area indicating the extent of cell calcification. **(B)** BMP-2 and RunX2 protein and mRNA expression using Western blotting and RT-PCR (n = 3, *p* < 0.05). **(C)** CD44 protein expression using Western blotting (n = 3, *p* < 0.05). **(D)** Calcium ion concentration in the supernatant using a calcium colorimetric assay kit (n = 3, *p* < 0.05). **(E)** TG2 protein and mRNA expression using Western blotting and RT-PCR (n = 3, *p* < 0.05). All comparisons were performed relative to the control group. Con, control; CM, calcium media. * denotes *p* < 0.05.

To confirm the EC calcification model, we utilized alizarin red staining, osteogenic protein expression, and calcium colorimetric assay after treating HUVECs with CM for 14 days Figure 1A shows a large deposition of an orange–red complex in the HUVEC cytoplasm, indicating the presence of calcium deposits in treated cells. An EndMT-related marker (CD44) was detected via Western blotting and significantly higher CD44 protein levels were observed in the CM group compared than in the control group (Figure 1B). Significantly higher expression of osteoblast differentiation-related protein BMP-2 and Runx2 at both the mRNA and protein levels was also observed in the CM group compared to the control group (Figure 1C). Compared with the control group, a significant increase in cell calcium concentration in the CM group was observed (CM: 5.01 ± 0.27 μg/μL), whereas the calcium ion concentration in the normal group was below the detection limit (Figure 1D). Finally, TG2 protein and mRNA expression were significantly increased in the CM group (Figure 1E). Altogether, these results demonstrated that high phosphate concentration can transform ECs into osteoblasts through EndMT.

### 3.2 Effect of TG2 expression and activity on EC calcification

**FIGURE 2:**
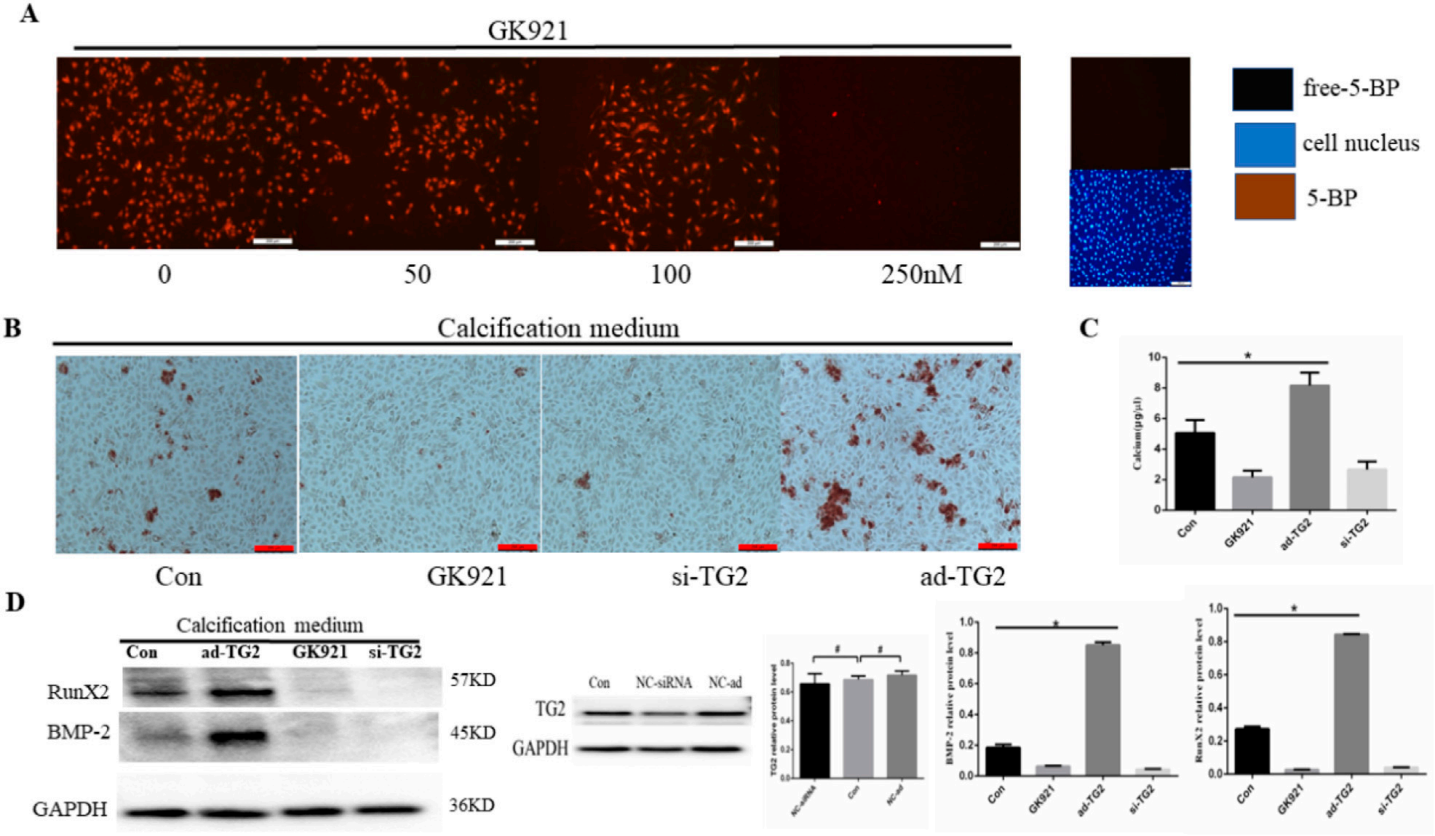
Effect of TG2 expression and activity on EC calcification. **(A)** Cells exposed to 5-BP (400 μM) for 4 h after 24 h of serum starvation before exposure to GK921. As a negative control, 5-BP treatment was omitted. Cells were exposed to GK921 at 50, 100, and 250 nM for 24 h and then stained with streptavidin-555-conjugated BP and 4′,6-diamidino-2-phenylindole (DAPI). The image shows the overlay of TG2 and 5-BP staining, indicating colocalization. **(B)** Alizarin red S staining detecting calcium salt deposits. The orange–red area indicates the region of cell calcification. **(C)** Calcium ion concentration in the supernatant was measured using a calcium colorimetric assay kit (n = 3, *p* < 0.05). **(D)** BMP-2 and RunX2 protein expression levels in ECs (n = 3, *p* < 0.05). Con, Control; si-TG2, silencing TG2 gene; ad-TG2, overexpressed TG2 gene; NC, negative control. All comparisons were performed relative to the control group. * denotes *p* < 0.05, ^#^ denotes *p* > 0.05.

Research has revealed that GK921, a TGM2-specific inhibitor, blocked mesenchymal transdifferentiation and showed significant therapeutic efficacy in a mouse model of glioma stem cells (Johnson et al., 2008). However, the role of GK921 in EC calcification has not yet been reported. Initially, the optimal concentration of GK921 for inhibiting TG2 activity in ECs was analyzed using the 5-BP probe method. At a concentration of 250 nM, TG2 activity in HUVECs was significantly inhibited by GK921 (Figure 2A). Subsequently, HUVECs with stably silenced or overexpressed TG2 gene were treated with CM for 14 days in the presence or absence of GK921 at 250 nM. In comparison to the control group, the group overexpressing TG2 gene exhibited a higher number of orange–red complexes in the cytoplasm of ECs, as presented in Figure 2B. No orange–red complexes were observed in the group with inhibited TG2 activity or in the group with silenced TG2 gene. To further investigate the effect of TG2 activity on calcium concentration, a calcium colorimetric assay kit was used to detect calcium concentrations in cell lysates for all four groups. The results demonstrated that compared with the control group, the group overexpressing the TG2 gene exhibited a significant increase in calcium concentrations (5.06 ± 0.49 vs*.* 8.17 ± 0.49 μg/μL, *p* < 0.05; Figure 2C). By contrast, calcium concentrations significantly decreased in the TG2 activity inhibition group (5.06 ± 0.49 vs*.* 2.15 ± 0.25 μg/μL, *p* < 0.05) and the TG2 gene silencing group (5.06 ± 0.49 vs*.* 2.67 ± 0.30 μg/μL, *p* < 0.05), as compared to the control group. Finally, the expression of BMP-2 and Runx2 proteins, which are related to osteoblast differentiation, was analyzed in all four groups. Figure 2D shows that compared to the control group, the group overexpressing the TG2 gene exhibited a significant increase in BMP-2 and Runx2 protein expression. Nevertheless, the TG2 activity inhibition group and the TG2 gene silencing group did not exhibit any detectable protein expression of BMP-2 and Runx2. These results collectively indicated that the activity of TG2 is a crucial factor in promoting EC calcification and that GK921 can effectively inhibit this process.

### 3.3 Effect of TG2 expression and activity on IL-6 secretion and NF-κB pathway

**FIGURE 3:**
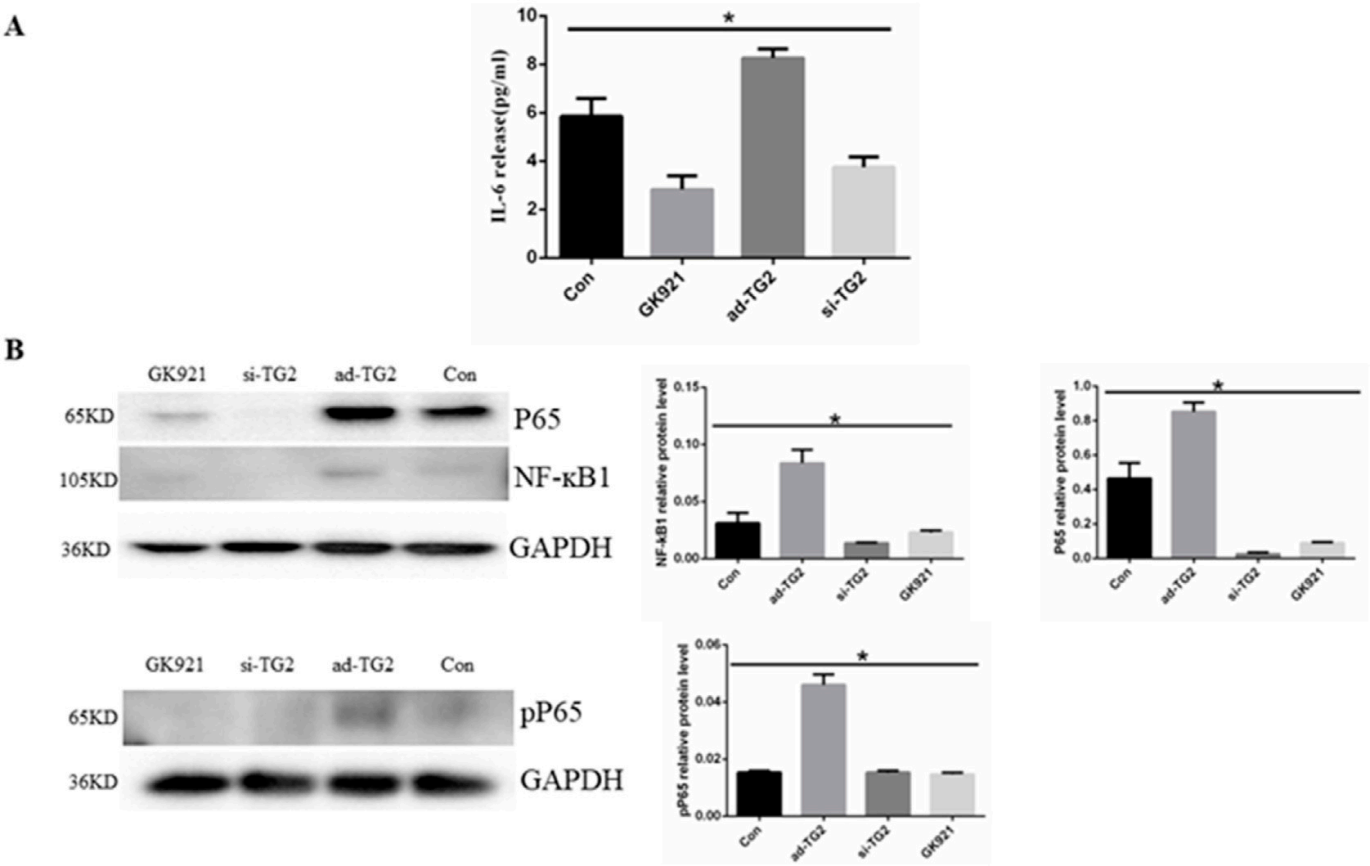
Effect of TG2 on IL-6 secretion and NF-κB pathway. **(A)** Quantification of IL-6 in the supernatant using ELISA (n = 3, *p* < 0.05). Cell supernatants were collected after the silencing or overexpression of TG2 gene, and ECs were treated with GK921 (250 nM) for 24 h **(B)** NF-ĸB, P65, and pP65 protein expression levels detected using Western blotting (n = 3, *p* < 0.05). Con, Control; si-TG2, silencing TG2 gene; ad-TG2, overexpressed TG2 gene; pP65, phosphotyrosine P65. All comparisons were performed relative to the control group. * denotes *p* < 0.05.

Previous research has demonstrated that TG2 mediates autocrine IL-6 signaling in various types of cells (Johnson et al., 2008; Jia et al., 2020; Zhang and McCarty, 2017). Thus, we aimed to investigate the effect of TG2 on IL-6 secretion in HUVECs. The amount of IL-6 in cell supernatants was assessed using ELISA. As depicted in Figure 3A, the overexpression of the TG2 gene resulted in a significant increase in IL-6 secretion compared to the control group, whereas the inhibition of TG2 activity and silencing of the TG2 gene (5.85 ± 0.42 vs. 3.75 ± 0.25 pg/mL, *p* < 0.05) led to a significant decrease in IL-6 secretion (5.85 ± 0.42 vs. 2.83 ± 0.32 pg/mL, *p* < 0.05 and 5.85 ± 0.42 vs. 8.26 ± 0.22 pg/mL, *p* < 0.05, respectively). The effect of TG2 on the NF-κB signaling pathway in ECs was subsequently investigated. The expression level of NF-κB1 and p65 proteins decreased upon silencing the TG2 gene or inhibiting TG2 activity, whereas overexpressing the TG2 gene increased their protein levels (Figure 3B). Moreover, measuring the levels of phosphotyrosine P65 (pP65) in the NF-κB signaling pathway revealed that TG2 gene overexpression may enhance the phosphorylation of P65 at the tyrosine residue. These findings suggest that TG2 activates the NF-κB signaling pathway, which subsequently regulates IL-6 autocrine signaling.

### 3.4 IL-6 activates JAK2/STAT3 signaling pathway to mediate EC autophagy

**FIGURE 4:**
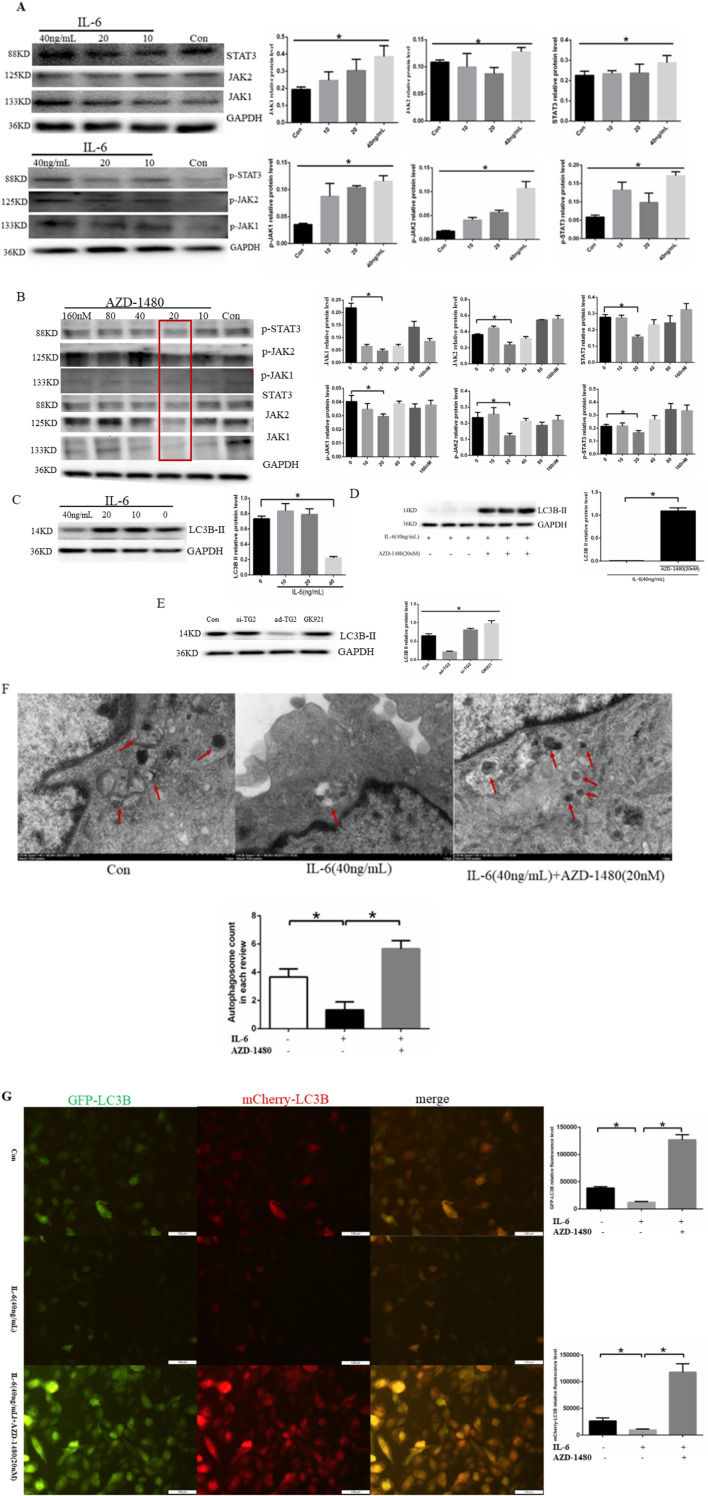
IL-6 inhibited autophagy in HUVECs via the activation of the JAK2/STAT3 signaling pathway. **(A)** Representative western blots showing that IL-6 promoted p-JAK1/p-JAK2/p-STAT3 expression in a concentration-dependent manner after HUVECs were treated with human recombinant IL-6 protein at the concentrations of 10, 20, and 40 ng/mL for 24 h (n = 3, *p* < 0.05). **(B)** Representative western blots showing that AZD-1480 inhibited P-JAK1/P-JAK2/P-STAT3 expression at 20 nM after HUVECs were treated with AZD-1480 for 24 h (n = 3, *p* < 0.05). **(C)** Representative western blots showing that 40 ng/mL IL-6 inhibited autophagy marker protein LC3B II expression (n = 3, *p* < 0.05). **(D)** AZD-1480 (20 nM) increased LC3B II protein expression (n = 3, *p* < 0.05). **(E)** Western blots showing LC3B II protein expression through TG2 gene overexpression or silencing or after GK921 treatment (n = 3, *p* < 0.05). **(F)** Autophagosomes in HUVECs with 40 ng/mL IL-6 along with or without AZD-1480 (20 nM) observed using a transmission electron microscope. **(G)** Quantitative analysis of mCherry-eGFP-LC3 in HUVECs after treatment with 40 ng/mL IL-6 along with or without AZD-1480 (20 nM). Con, Control; AZD, AZD-1480. All comparisons were performed relative to the control group. * denotes *p* < 0.05.

To examine whether IL-6 regulated EC autophagy through JAK2/STAT3 signaling, HUVECs were treated with human recombinant IL-6 protein at the concentrations of 10, 20, and 40 ng/mL for 24 h. The findings indicated that IL-6 induced a concentration-dependent increase in the expression of JAK1/p-JAK1, JAK2/p-JAK2, and STAT3/p-STAT3 protein expression (Figure 4A). Based on the fact that AZD-1480 is a JAK1/2 inhibitor, we hypothesized that the inhibitory effect of AZD-1480 on the autophagy of ECs is mediated via STAT3 activation. To examine the interaction among AZD-1480, IL-6, and EC autophagy, the expression of the JAK2/STAT3 pathway components was analyzed. In line with our hypothesis, Western blot revealed that 20 nM AZD-1480 treatment reduced the expression of P-JAK2 and P-STAT3 proteins when compared with the vehicle control (Figure 4B). The effect of IL-6 on EC autophagy was additionally examined, as shown in Figure 4C. At 40 ng/mL, IL-6 protein significantly suppressed LC3B-II protein expression (Figure 4C), whereas AZD-1480 (20 nM) increased LC3B-II protein expression (Figure 4D). The analysis of the effect of TG2 on the LC3B-II protein expression showed that LC3B-II protein expression was increased when the TG2 gene was overexpressed and LC3B-II protein expression was decreased when the TG2 gene was silenced or inhibited using the TG2 inhibitor GK921 (Figure 4E). In short, 40 ng/mL IL-6 and TG2 overexpression exhibited consistent effects on EC autophagy (Figures 4D, E). To visualize autophagosomes in the ECs of the control group, IL-6 (40 ng/mL) group, and IL-6 (40 ng/mL) with AZD-1480 (20 nM) group, transmission electron microscopy was employed. In the control group, a moderate number of autophagosomes were observed in HUVECs, whereas in the IL-6 (40 ng/mL) group, the number of autophagosomes was significantly decreased (Figure 4F). However, compared with the IL-6 (40 ng/mL) group, the administration of AZD-1480 (20 nM) significantly increased the number of autophagosomes (Figure 4F). The ECs were first transfected with Cherry-eGFP-LC3 adenovirus, before being treated with 40 ng/mL IL-6 for 24 h. The fluorescence level of Cherry-eGFP-LC3 was detected in transfected ECs with or without the addition of AZD-1480 (20 nM). Compared with the normal group, 40 ng/mL IL-6 reduced the expression of LC3B in ECs, whereas AZD-1480 (20 nM) significantly increased the expression of LC3B (Figure 4G). In summary, the results suggest that IL-6 inhibits autophagy in HUVECs by activating the JAK2/STAT3 signaling pathway.

### 3.5 Effect of autophagy on cell calcification

**FIGURE 5:**
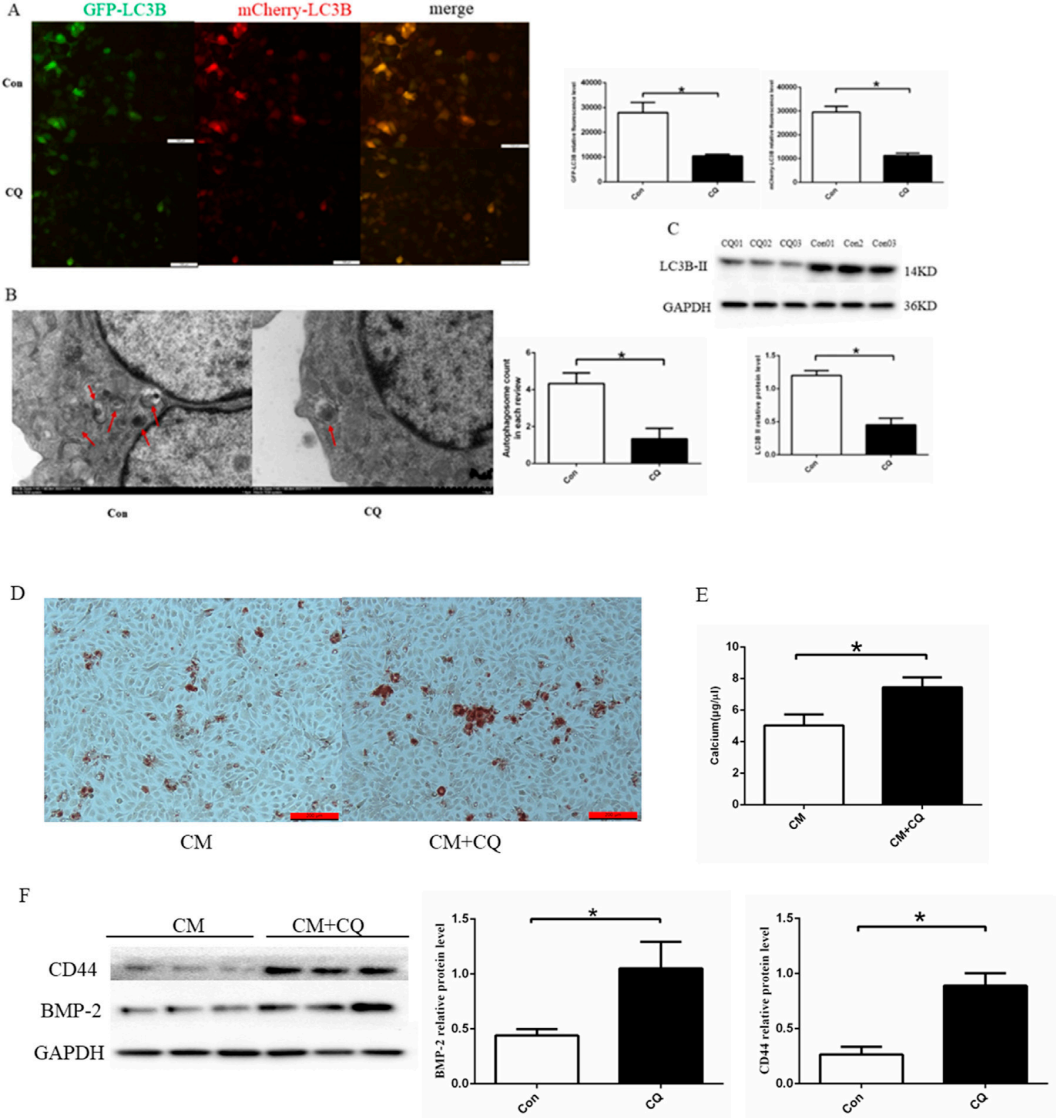
Effect of autophagy on cell calcification. **(A)** Quantitative analysis of Cherry-eGFP-LC3 in HUVECs after treatment with 20 μM chloroquine phosphate (n = 3, *p* < 0.05). **(B)** Autophagosomes in HUVECs with 20 μM chloroquine phosphate observed using a transmission electron microscope (n = 3, *p* < 0.05). **(C)** chloroquine phosphate decreased LC3B II protein expression (n = 3, *p* < 0.05). **(D)** Alizarin red S staining detecting calcium salt deposits. The orange–red area indicates the region of cell calcification. **(E)** Calcium ion concentration in the supernatant was measured using a calcium colorimetric assay kit (n = 3, *p* < 0.05). **(F)** BMP-2 and CD44 protein expression using Western blotting (n = 3, *p* < 0.05). CM, calcium media; CQ, chloroquine phosphate. All comparisons were performed relative to the control group. * denotes *p* < 0.05.

We used Chloroquine phosphate (autophagy inhibitor) to further analyze the effect of autophagy on endothelial cell calcification. HUVECs were treated with chloroquine phosphate(20 μM) for 24 h. The findings indicated that chloroquine phosphate induced an obvious decrease in the expression of LC3B-Ⅱ、autophagy fluorescence and the number of autophagosomes(Figures 5A–C). Then, we established EC calcification model. To confirm the EC calcification model, we utilized alizarin red staining, osteogenic protein expression, and calcium colorimetric assay after treating HUVECs with CM with or without chloroquine phosphate(20 μM) for 14 days. Alizarin red S staining revealed that chloroquine phosphate promoted calcium salt deposition in the cells (Figure 5D). In addition, cells treated with chloroquine phosphate demonstrated a significant increase in intracellular calcium ion concentration compared to the CM group (5.03 ± 0.70 μg/μL vs. 7.44 ± 0.64 μg/μL, *p* < 0.05) (Figure 5E). Finally, we assessed the expression of BMP-2 and CD44, as shown in Figure 5F. The results demonstrated that chloroquine phosphate promoted BMP-2 and CD44 proteins expression, compared to the CM group. Based on the results, it can be concluded that inhibition of autophagy can promote cell calcification.

### 3.6 Effect of AZD-1480 on cell calcification

**FIGURE 6:**
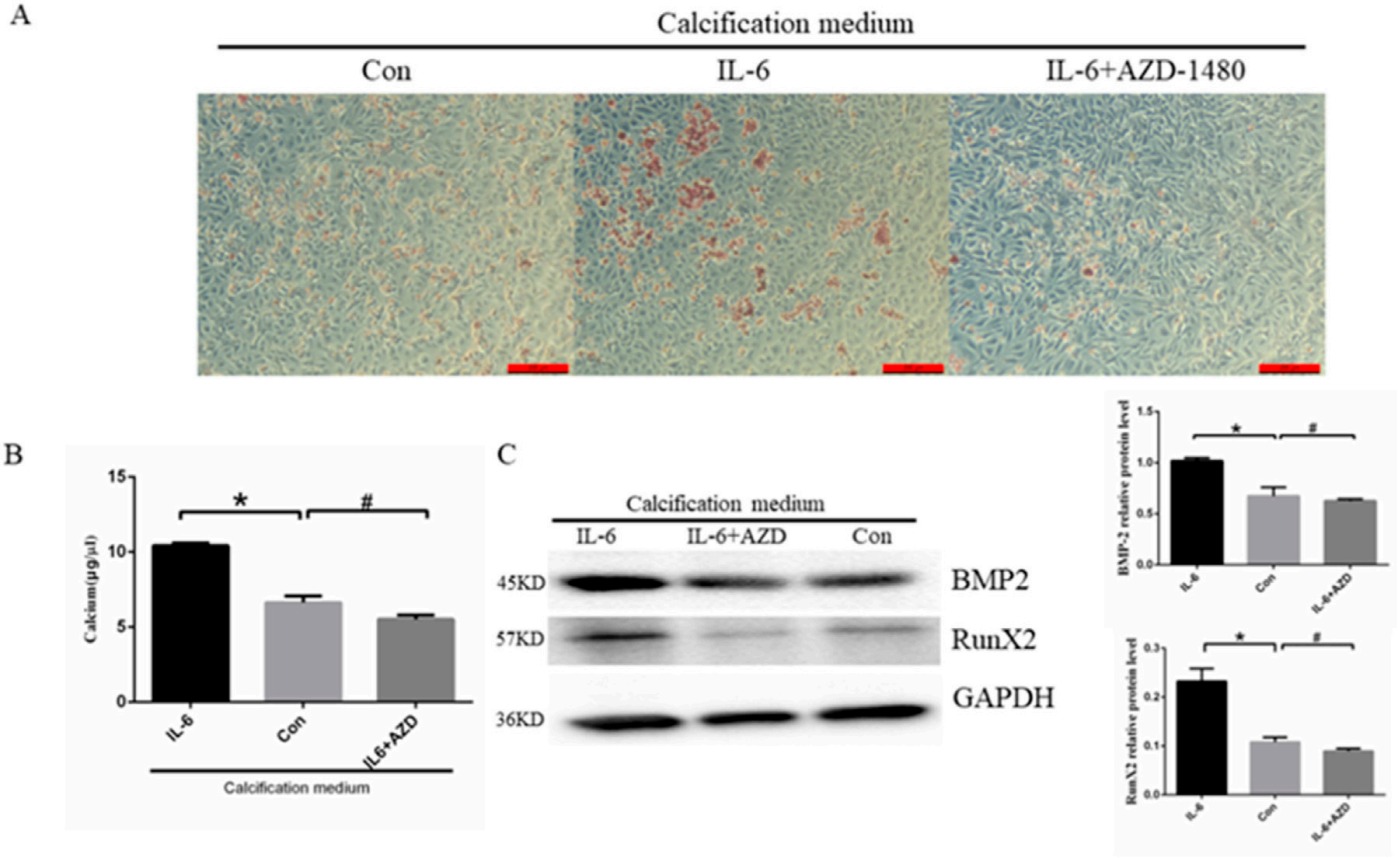
Treatment with AZD-1480 inhibited cell calcification. **(A)** Alizarin red S staining detecting calcium salt deposits. The orange–red area represents the location of cell calcification. **(B)** Calcium ion concentration in the supernatant was measured using a calcium colorimetric assay kit (n = 3, *p < 0.05, #*p* > 0.05). **(C)** RunX2 and BMP-2 expression levels in HUVECs treated with IL-6 and/or AZD-1480, as measured via Western blot analysis (n = 3, *p < 0.05, #*p* > 0.05). Con, Control; AZD, AZD-1480. All comparisons were performed relative to the control group. * denotes *p* < 0.05, # denotes *p* > 0.05.

It is well established that the proinflammatory cytokine IL-6 plays an important role in the development of atherosclerosis. As demonstrated by previous experiments, AZD-1480 can promote IL-6-induced autophagy. To further investigate the impact of inhibiting autophagy on EC calcification, the cells were treated with CM containing IL-6 (40 ng/mL) with or without AZD-1480 (20 nM) for a period of 14 days. Alizarin red S staining revealed that IL-6 promoted calcium salt deposition in the cells, whereas treatment with AZD-1480 inhibited this effect (Figure 6A). In addition, cells treated with IL-6 demonstrated a significant increase in intracellular calcium ion concentration compared to the control group (6.63 ± 0.26 μg/μL vs*.* 10.42 ± 0.09 μg/μL, *p* < 0.05). Conversely, cells treated with AZD-1480 showed a trend of decreased intracellular calcium ion concentration, although this effect was not statistically significant (6.63 ± 0.26 μg/μL vs. 6.52 ± 0.28 μg/μL, *p* > 0.05) (Figure 6B). Finally, we assessed the expression of BMPs, as shown in Figure 6C. The results demonstrated that IL-6 promoted calcium deposition in ECs, as indicated by the increased expression of RUNX2 and BMP-2 proteins, compared to the control group. Conversely, treatment with AZD-1480 inhibited the expression of RunX2 and BMP-2 proteins, suggesting that it exhibits a protective effect against EC calcification. Based on the results, it can be concluded that IL-6 promoted calcium deposition in ECs, whereas AZD-1480 inhibited EC calcification.

## 4 Discussion

ECs comprise the inner layer of blood vessels and are known to be directly affected by serum stimuli such as glucose, inflammatory factors, phosphorous, and hemodynamics. While VSMC-related lineages have also been implicated in the MGP-deficient and atherosclerotic models of VC, the potential contribution of endothelium has been highlighted in the atherosclerotic and diabetic models of VC (Speer et al., 2009; Yao et al., 2013). At present, many studies have focused on the role of VSMCs on VC (Durham et al., 2018; Jaminon et al., 2019; Chen WR. et al., 2019; Cao et al., 2020) while ignoring the role of ECs on VC, particularly atherosclerotic calcification. ECs are not only critical for atherogenesis but also believed to be a source of osteoprogenitor cells that initiate intimal calcification. Therefore, our research investigated the molecular mechanism of EC calcification, which can provide new insights into the pathogenesis of atherosclerosis. Reports have suggested that ECs harbor osteogenic and chondrogenic potential and can contribute to heterotopic ossification in other tissues because of aberrant BMP signaling (Wylie-Sears et al., 2011; Dudley et al., 2008; Medici et al., 2010). Taken together, these data suggest that BMP signaling exerts some of its pro-osteogenic and inflammatory effects in the vasculature by acting on the endothelium and directly affecting the plasticity of those lineages (Yung et al., 2015).

The ability of endothelial lineages to develop the characteristics of the mesenchyme was first discovered in the developing heart, where endocardium transdifferentiates to form the atrioventricular cushion via EndMT (Camenisch et al., 2002; Liebner et al., 2004; Nakajima et al., 2000). However, ECs lose their ability to adhere and undergo a cytoskeletal change that results in a long spindle-shaped morphology, whereas newly formed mesenchymal cells have enhanced invasion and migration abilities (Yuan et al., 2021). Upon EndMT, ECs acquire multiple differentiation potentials and differentiate into osteoblasts, chondrocytes, and adipocytes, among others, under the appropriate conditions (Medici and Kalluri, 2012; Yu et al., 2014). At the same time, we demonstrated that ECs were transformed into osteoblasts through EndMT after they were treated with CM and BMP-2 protein in this study.Research has suggested the involvement of TG2 in regulating cancer cell autophagy. Increasing evidence has demonstrated that TG2 is closely associated with constitutive nuclear factor-kappa B (NF-κB) expression in cancer cells (Kumar and Mehta, 2012; Brown, 2013). Moreover, numerous studies have shown that TG2 is associated with inflammation and the NF-κB pathway, making it a potential therapeutic target for various diseases (Li et al., 2023; Szondy et al., 2017). For example, the increased expression of TGase2 (TG2) and subsequent activation of NF-κB has been shown to contribute to drug resistance in breast cancer cells independent of EGF signaling (Kim et al., 2006). In a previous study, a positive regulatory loop was found among TGM2 (TG2)-NF-ĸB/NF-κB signaling, IL-6, and autophagy in cancer, as exemplified by mantle cell lymphoma as the model system in that study (Zhang and McCarty, 2017). Additionally, research has shown evidence for the existence of a TG2/NF-κB signaling loop in breast cancer, the nature of the signal transduction that activates this loop, and phenotypic consequences stemming from its aberrant activation (Brown, 2013). TG2 forms complexes with NF-kB components, which drives the translocation of NF-kB to the nucleus and constitutive expression of NF-kB in mantle cell lymphoma progression (Jung et al., 2012). The interplay between autophagy and VC is complex and influenced by factors such as disease progression, anatomical site, and the local microenvironment. Autophagy activation in response to cellular damage is generally considered a protective mechanism, whereas in normal cells dysfunctional autophagy can lead to apoptotic activation (Zhou et al., 2021; Phadwal et al., 2020). However, current research has mainly focused on the role of autophagy in VSMCs with regard to VC, with limited studies on the involvement of ECs. It is widely accepted that ECs participate in VC through EndMT. Resveratrol was recently demonstrated to attenuate endothelial cell inflammation via induction of autophagy (Chen et al., 2013). Similarly, vitamin D was recently shown to elicit cytoprotective effects in the endothelium via augmenting autophagic flux (Uberti et al., 2014). Endothelial cells exposured in culture to oxidized lipoproteins or advanced glycation end-products induces autophagy, which was protective against endothelial cell injury (Schrijvers et al., 2011). When autophagy is inhibited, ECs are dysfunctional and differentiate into osteoblast-chondrogenic phenotype, which promotes calcification (Hill and Salabei, 2013). Our results also demonstrated that inhibition of autophagy promoted endothelial cell calcification through EndMT. The well-known proinflammatory factor IL-6 has been extensively researched for its role in regulating cell autophagy, but IL-6-mediated autophagy and its involvement in VC are not well understood (Hu et al., 2021; Rong et al., 2021; Takagaki et al., 2020; Pinto et al., 2022; Chen R. et al., 2019; Lu et al., 2018). The present research revealed that TG2 can regulate IL-6 autocrine signaling in ECs. Studies have shown that the NF-κB/IL-6 signaling pathway plays an important role in inflammation, immune responses, cell cycle regulation, and cell survival (Zheng et al., 2022; Alaaeldin et al., 2022). Likewise, the current research demonstrated that TG2 activated the NF-κB signaling pathway in ECs and proposes that TG2 promotes IL-6 autocrine signaling by activating the NF-kB signaling pathway in HUVECs, which represents a novel finding. Additionally, using the JAK1/JAK2 inhibitor AZD-1480, this study demonstrated that IL-6 inhibited HUVEC autophagy through the JAK2/STAT3 signaling pathway. AZD-1480 has been shown to inhibit tumor cell growth. For example, the combination of AZD1480 and 4-OH-TAM resulted in a significant suppression of breast cancer growth (Ng et al., 2022). Finally, functional rescue experiments with AZD-1480 resulted in the inhibition of HUVEC calcification. In conclusion, this research is the first to demonstrate the mechanism by which the TG2-IL-6-JAK2/STAT3 signaling axis regulated EC calcification through autophagy. However, the research has certain limitations. We could not validate the role of the TG2-IL-6-JAK2/STAT3 signaling axis through *in vivo* experiments due to limited resources. Moreover, sites where TG2 binds to the NF-κB pathway proteins were not identified. Taken together, the present study findings provided novel insights into the mechanism underlying the regulation of EC calcification in atherosclerotic calcification, offering new possibilities for understanding the pathogenesis of atherosclerosis and presenting a potential therapeutic target for treating atherosclerosis.

## 5 Conclusion

In summary, our findings demonstrated that CM increased TG2 activity and expression, which activated the NF-κB signaling pathway, and induced IL-6 autocrine signaling in ECs. Furthermore, IL-6 activated the JAK2/STAT3 signaling pathway to suppress cell autophagy and promoted ECs calcification. Our findings provided new evidence that TG2 regulated vascular calcification.

## Data Availability

The original contributions presented in the study are included in the article/supplementary material, further inquiries can be directed to the corresponding author.
